# A Modified Amino Acid Network Model Contains Similar and Dissimilar Weight

**DOI:** 10.1155/2013/197892

**Published:** 2013-01-02

**Authors:** Xiong Jiao, Lifeng Yang, Meiwen An, Weiyi Chen

**Affiliations:** ^1^Institute of Applied Mechanics and Biomedical Engineering, Taiyuan University of Technology, Taiyuan 030024, China; ^2^College of Computer Science and Technology (College of Software), Taiyuan University of Technology, Taiyuan 030024, China

## Abstract

For a more detailed description of the interaction between residues, this paper proposes an amino acid network model, which contains two types of weight—similar weight and dissimilar weight. The weight of the link is based on a self-consistent statistical contact potential between different types of amino acids. In this model, we can get a more reasonable representation of the distance between residues. Furthermore, with the network parameter, average shortest path length, we can get a more accurate reflection of the molecular size. This amino acid network is a “small-world” network, and the network parameter is sensitive to the conformation change of protein. For some disease-related proteins, the highly central residues of the amino acid network are highly correlated with the hot spots. In the compound with the related drug, these residues either interacted directly with the drug or with the residue which is in contact with the drug.

## 1. Introduction

In living cells, proteins are very important molecules, and they participate in almost all of the cell functions. During these biological activities, the structure of some proteins shows an obviously conformational flexibility. For a correct and fast implementation of the biological functions through the conformation change, there needs a motor coordination for the residues in different parts of the protein. In this process, a fast communication mechanism is vital for the information sharing between residues about these concerted actions. In fact, this information exchange is achieved through the interaction between residues. But when we put all these residues and the interactions between them together, the protein becomes a very complicated system. 

On the other hand, from the viewpoint of complex network [1, 2], a protein molecule can be treated as a complex network. In this network, each residue can be simplified as a node, and the interaction between different residues is treated as the link. With this useful tool—complex network, some new research ideas and methods are applied to the study of the structure-function relationship, and some phenomenon can be explained through the analyzing of this network. Such related work as the identification of the “key residues” through the network parameter—betweenness [3]. In the measuring process of the topology of the protein contact network, the result shows that the kinetic ability for folding is determined by the topological properties of the protein conformation [4]. Through the biological networks, the rigidity and flexibility of protein structure can be analyzed. Furthermore, with this approach, the cytoskeletal tensegrity can be discussed [5]. The network model also has been wildly used in the drug design and drug discovery [6].

In the amino acid network, each residue is simplified to a single point, and this point is used as the network node. Generally, the carbon alpha is selected as the network node. In some other network models, a point between the carbon alpha and the carbon beta is used to as the network node. The links between these nodes are determined by the distance between them. If the distance between two nodes is less than a cut-off value, then there will exist a link between these two nodes. This cut-off is usually set to 7.0 angstrom [7] or set to 8.5 angstrom [3]. 

There is another type of amino acid network model. In this model, each residue is also simplified to a node. But the link between two nodes is based on the atom contact between these two residues. A cut-off value—4.5 angstrom [8], or 5.0 angstrom [9], is used as a criterion for the contacts between atoms. If there is an atom contacts between two residues, these two nodes will be connected by a link. For different amino acid network models, the criterion to dictate residue contacts has been reviewed and analyzed [10]. In this paper, the Miyazawa-Jernigan potential is used to construct the link weight, so the side chain center is used to represent the node, and the cut-off value used by Miyazawa and Jernigan is also used in this work [11, 12]. 

In the weighted amino acid network, in which the link is based on a contact between different residues, the weight of the link can be drawn from the contact probability between different residues [3], or the weight can be drawn from a statistical residue contact potential [11–13]. With the contact potential as the link weight, a weight elastic network model is used to calculate the protein structure dynamics [13]. For the network model based on atom contact, the weight of the link can be deduced from the number of atom contacts between nodes. Furthermore, when the diversity of amino acids is taken into account, these weights can be modified by a normalization factor [8]. 

For the weight of the link, it can be classified into two types. One is the similar weight and the other is the dissimilar weight [14]. For the similar weight, the value describes the similarity between two nodes. A higher value means that the two nodes are more similar, and the distance between them will be shorter. As for the dissimilar weight, a higher weight value, corresponding to a longer distance between the two nodes, means that the difference between these two nodes are more distinct.

For the weighted amino acid network, the related research work is underway, and many questions needed to be explored, such as which parameter should be selected as the weight and how to assign the weight to the link with a more reasonable mode. In our previous work, we proposed a weight amino acid model [15], but only one type of weight—similar weight is used in the previous model, so we cannot get a more detailed description of the interaction situation between residues. 

This paper will modify the previous model with two types of weight, and the weight used in this paper is based on a self-consistent statistical contact energy between residues [12]. In this paper, firstly, the construction methods of the weighted network are compared. Then, for the 197 proteins with low homology, the weighted amino acid networks are constructed and the statistic characteristics of the parameters of these networks are studied, including the average clustering coefficient (*C*) and the average shortest path length (*L*). Thirdly, with this weighted network, in order to get a relation between the change of the network parameter and the change of the protein conformation, we studied the changes of the average shortest path length for the small protein CI2 on its high temperature unfolding pathway. The last, take the FKBP-FK506 as an example, we show the application of amino acid network in the drug design. 

## 2. Theory and Method

In this weighted amino acid network, for each amino acid, the geometrical center of the side chain is chosen to represent the network node. The link between a pair of nodes is determined by the distance between them. If the distance between residues *i* and *j* (marked with *r*_*ij*_) is less than the cut-off (*r*_*c*_), there will be a link between them. In this paper, the cut-off is 6.5 angstrom. Thereby, the adjacency matrix element of the unweighted amino acid network can be expressed as follows:

(1) $$\begin{array}{c} a_{ij}=\{\begin{array}{cc} 1, & i\neq j,\;\;r_{ij}<r_{c},\\ 0, & i=j\;\;\text{or}\;\;r_{ij}\geq r_{c}. \end{array} \end{array}$$

Based on the contact potential between residues, the weighted network can be constructed. In the previous model, we use another set of contact potential. All the items of the contact potential are less than zero, and the calculation of the repulsive interaction between residues is very complex. 

In this work, we adopt a self-consistent interresidue contact potential to construct the weight of the link. In this contact potential, if two residues are attracted in most cases, the potential between them will get a negative value, and if they are repulsed generally, the potential will be a positive value. With this contact potential, the adjacency matrix element of the weighted amino acid network can be expressed as

(2) $$\begin{array}{c} a_{ij}^{w}=\{\begin{array}{cc} a_{ij}w_{ij}, & j\neq i\pm 1,\\ 0, & j=i\pm 1. \end{array} \end{array}$$

In this definition, we take the contact potential between residues *i* and *j* as the link weight, marked as *w*_*ij*_. The value of *w*_*ij*_ is related to the types of the residues *i* and *j*. For the covalent bond between residues *i* and *i* ± 1, the link weight is assumed as zero. 

 In this amino acid network, if the two nodes are attracted, the potential between them is a negative real number, so, the link between them will get a negative weight. If the attraction between these two nodes become stronger, the absolute value of the weight will become a bigger one. Then, the negative weight can be treated as a similar weight. For the same reason, if the two nodes are repulsed, the potential between them corresponds to a positive real number, and the link between them will get a positive weight. When the repulsion between these two nodes become stronger, the link will get a bigger positive weight value. So, the positive weight can be treated as a dissimilar weight.

Thus, based on the weighted adjacency matrix, the distance matrix can be constructed and the definition of its element can be written as follows. We labeled this definition as definition 1:

(3) $$\begin{array}{c} d_{ij}=\{\begin{array}{cc} 0, & i=j,\\ \infty , & a_{ij}=0,\;\;i\neq j,\\ \frac{1}{\left(1-w_{ij}\right)}, & w_{ij}<0,\;\;a_{ij}=1,\\ 1, & w_{ij}=0,\;\;a_{ij}=1,\\ 1+w_{ij}, & w_{ij}>0,\;\;a_{ij}=1. \end{array} \end{array}$$

When the interaction between two residues is an attractive interaction, the corresponding link weight is a similar one. In this distance definition, a reciprocal function of the weight is used to represent the distance between a pair of attracting nodes. For a stronger attractive interaction between residues, the actual distance between them is shorter than others. And because the weight for attraction is negative, a bigger absolute value corresponds to a shorter distance, as defined in the distance matrix.

At the same time, if the interaction between residues is repulsive, the corresponding link weight is a dissimilar one. The distance definition between them is a linear combination function of the weight. A stronger repulsive interaction, corresponding to a longer actual distance between them, will get a bigger distance value from the distance matrix.

The network model used in this work is an undirected model, and the link is just to represent the existence of the interaction between these two residues. The status of the two ends of a link is equal. So, for the weighted network and the unweighted one, the adjacency matrixes are all symmetric matrixes. In the distance matrix, the similar weight and the dissimilar weight are coexistent in the same distance matrix, and the distance matrix is also a symmetric one.

For a comparison between different definitions, if we do not make a difference between the similar weight and the dissimilar one, and just the dissimilar weight is used in this model, the distance matrix can be defined as below. We labeled it as definition 2:

(4) $$\begin{array}{c} d_{ij}=\{\begin{array}{cc} 0, & i=j,\\ \infty , & a_{ij}=0,\;\;i\neq j,\\ 1+\frac{w_{ij}}{2.19}, & a_{ij}=1. \end{array} \end{array}$$

On the other hand, we can convert the dissimilar weight to the similar weight. The distance between nodes can be defined as below, and it is labeled as definition 3:

(5) $$\begin{array}{c} d_{ij}=\{\begin{array}{cc} 0, & i=j,\\ \infty , & a_{ij}=0,\;\;i\neq j,\\ \frac{1}{\left(1-w_{ij}\right)}, & a_{ij}=1. \end{array} \end{array}$$

Additionally, a new network parameter—strength—is introduced into the weighted amino acid network. The strength of node *i* can be written as [16, 17]

(6) $$\begin{array}{c} S_{i}=\sum_{j=1}^{N}\left|a_{ij}^{w}\right|, \end{array}$$

where *N* is the number of network nodes and *a*_*ij*_^*w*^ is an element of the weighted adjacency matrix. 

The clustering coefficient of the weighted network can be calculated using the next expression [16, 17]:

(7) $$\begin{array}{c} C_{i}=\frac{1}{S_{i}\left(K_{i}-1\right)}\sum_{j,h}a_{ij}a_{ih}a_{jh}\frac{\left|w_{ij}\right|+\left|w_{ih}\right|}{2}, \end{array}$$

where *S*_*i*_ is the strength of the node *i*, and *K*_*i*_ is its degree. The means of *a*_*ij*_ and *w*_*ij*_ are the same as that of the expression (2).

The betweenness of node *u* can be defined as below [18]:

(8) $$\begin{array}{c} B_{u}=\sum_{i,j}\frac{\sum_{l\in S_{ij}}\delta_{l}^{u}}{\left|S_{ij}\right|}. \end{array}$$

The denominator is the number of shortest paths between *i* and *j*, and the numerator is the number of shortest paths between *i* and *j* through node *u*. Betweenness is a useful measure of the node's importance to the network. In order to reflect the significance of betweenness for different nodes, the *Z*-score is introduced, and the definition of *Z*-score for *B*_*u*_ is as follows [19]:

(9) $$\begin{array}{c} Z_{u}=\frac{B_{u}-\bar{B}}{\sigma }, \end{array}$$

where *B*_*u*_ is the betweenness of residue *u*, $\bar{B}$ is the average value of the betweenness of all protein residues, and *σ* is the standard deviation of these betweenness values.

## 3. Results and Discussion

### 3.1. Comparison between Different Definitions of the Distance

**Figure 1:**
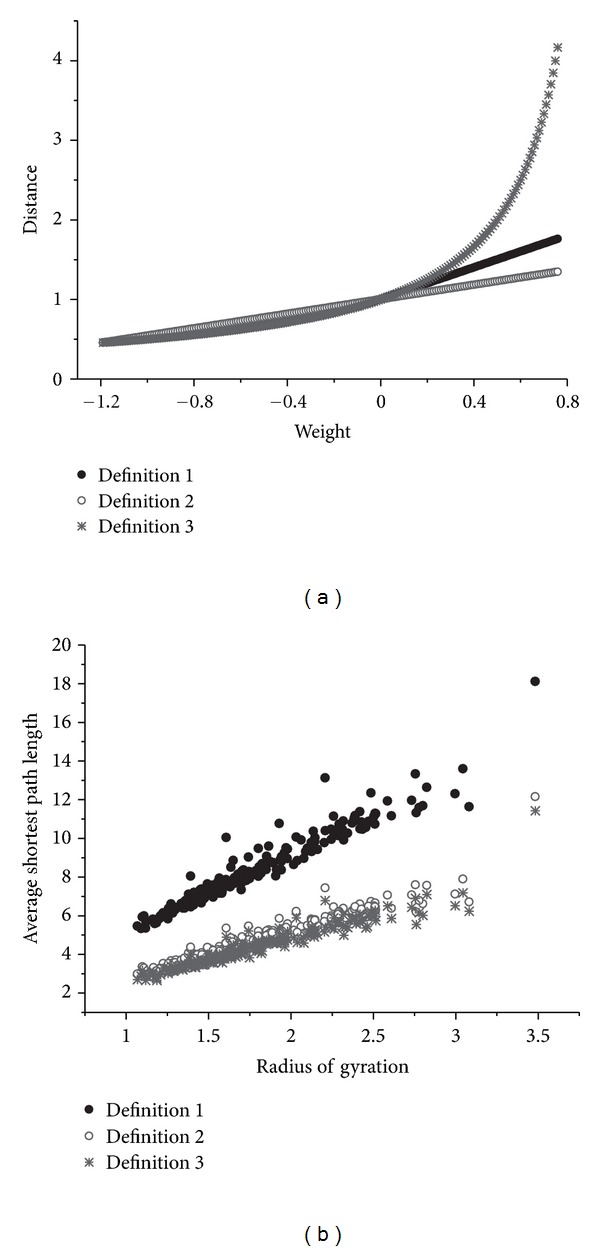
The comparison between the three definitions of the distance matrix. (a) The relation between the weight and the distance. (b) The comparison of the correlation between the average shortest path length and the radius gyration.

For the contract potential used to construct the weighted network in this paper, the value ranges from −1.19 to 0.76. The corresponding distance for varying weights, get from the three different definitions of distance matrix, is shown in Figure 1(a). From this figure, we can see that, when the interaction between residues is a repulsive interaction, if the link weight is a similar weight, the distance got from the distance definition 3 will increase sharply. But based on common sense, it is unreasonable. 

On the other hand, in the statistical calculation process of this self-consistent statistical contact potential between different types of amino acids, the cut-off is 6.5 angstrom, and this cut-off is still being used in the contact definition between residues in this paper. So, the distance between a pair of network nodes should be less than 6.5 angstrom. In a statistic calculation process of the actual distance between nodes, the result shows that this actual distance ranges from 3.88 to 6.5 angstrom. The ration of the maximum with the minimum is about 1.7. In the definition 3, due to the sharply increasing of the distance, this ratio is about 9. But for definition 1 and 2, this ration is about 3. So, it can be concluded that in the definition 3, it is not a reasonable assumption that the positive weight be treated as a similar weight. 

In our previous work, there is only one type of weight—similar weight. This definition should be revised as follows: a link with a positive weight should be assigned a dissimilar weight, as the rule of definition 2.

At the same time, in the statistic calculation process of the actual distance between nodes, as mentioned above, the result shows that the a great majority of the distances is about 5 angstrom, and most of the interactions between these nodes are an attractive one. So, the middle part of the weight-distance curve should be a nearly horizontal line. For the negative weight, the curve of definition 3 is more horizontal than that of definition 2. This phenomenon shows that when the link weight is a negative value, the similar weight assumption is more suitable to reflect the truth. 

Based on the above discussion, we can see that the similar weight assumption is reasonable for a negative weight, and the dissimilar weight assumption is suitable for a positive value. Put all these together, we can get definition 1, and the following calculation of distance will use the definition showed in (3).

With a set of 197 proteins selected from the Protein Data Bank (PDB), the weighted amino acid networks are constructed. These proteins include the four structure types: *α*, *β*, *α* + *β*, and *α* − *β*. The resolution of these selected proteins is better than 1.8 Å and the sequence identity is less than 20%. The sizes of proteins vary from 51 to 779 residues. The distance matrix is calculated with definition 1.

Radius of gyration is a useful parameter to indicate the size of a molecule. With the network model, the average shortest path length can also be used as an indicator of the molecular size. For the data set, we calculate the radius of gyration for each protein with GROMACS [20]. At the same time, we can get the average shortest path length from the weighted amino acid network. The relation between the average shortest path length with the radius of gyration is shown in Figure 1(b). The correlation coefficient for the path length from definition 1 with the radius of gyration is 0.96. This correlation coefficient is 0.95 for definition 2 and 0.79 for definition 3. Definition 1 gets the best result.

### 3.2. The Small-World Characteristic of the Amino Acid Network

The “small-world” property is a very important character for complex networks, and the “small-world network” is ubiquitous in the real life, such as the neural networks [21, 22] and the gene network [23, 24]. A vivid example of the “small-world network” is the “six degrees separation” [21, 25]. In a small-world network, most nodes are not connected directly by a link. But due to the short-cut between nodes, most nodes can be reached from every other through a small number of steps. With the increasing of the nodes number, the shortest-path distance between nodes grows sufficiently slowly, and it can be expressed as a function of the logarithm of the number of nodes in the network.

For a complex network and a random network, if they have the same node numbers and the same link numbers, when some condition be satisfied, the complex network can be thought that it holds the “small-world” property. These conditions include two items, the first one is that the average clustering coefficient *C* of the complex network is far more than that of the random network, and the second condition is that the average shortest path length *L* is about equal to that of the random network. These conditions can be showed as the following expression [21]:

(10) $$\begin{array}{c} C\gg C_{r},\;\;L\geq L_{r}. \end{array}$$

In this inequality, *C*_*r*_ and *L*_*r*_ are the network parameter of the random network. *C*_*r*_ is the average clustering coefficient and *L*_*r*_ is the average shortest path length. *C*_*r*_ and *L*_*r*_ can be calculated with the following expressions [21]:

(11) $$\begin{array}{c} C_{r}\approx \frac{\langle K\rangle }{N},\;\;L_{r}\approx \frac{\ln N}{\ln\langle K\rangle }. \end{array}$$

In this expression, *N* is the node number and 〈*K*〉 is the average degree of the random network.

In the “small-world” network, most of the nodes can be reached fast from every other through the “short-cut” between residues. So, the average clustering coefficient of the network will get a relatively large value, and the average shortest path length (also be called: characteristic path length) will keep as small as that of a random network. 

**Figure 2:**
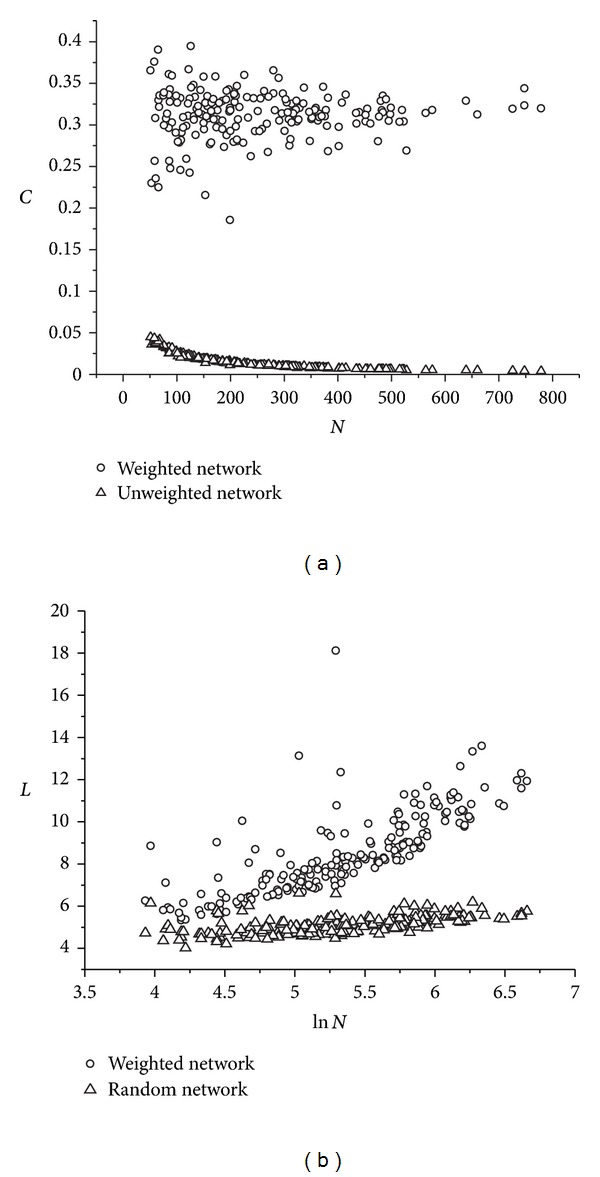
For the 197 proteins and the corresponding random networks with the same size, the comparison of network parameter. (a) The clustering coefficient of the weighted amino acid network and that of the random network with the same size; (b) the average shortest path length of the weighted amino acid network and that of the random network with the same size.

For the 197 proteins, we constructed the weighted network and calculated the average clustering coefficients and the average shortest path lengths with the distance matrix definition 1. Figures 2(a) and 2(b) showed these results. At the same time, for the random networks with the same size, these two parameters are calculated and the results are also shown in Figures 2(a) and 2(b). From these two figures, we can see that the weighted amino acid networks, contain similar and dissimilar weight for the link, present an obvious “small-world” property. From other works, we have known that the amino acid network is a “small-world” network, so, these results prove that the distinction introduced between similar and dissimilar weights is reasonable, and the construction method of the weighted network also is rational.

In the amino acid network, very few residues can get a high degree value. They usually lie in the core of the globular protein and act as the hubs of the networks [8, 26]. There are more interactions between these hub residues with other residues, so these hub residues play a vital role to the stability of whole protein structures [7, 8, 27]. In some other work, in order to embody the influence of the local environment, the distribution of residue clusters has been analyzed, and the outcome is a log-normal distribution [28]. 

### 3.3. The Change of Average Shortest Path Length with the Conformation Change

For exploring the changes of network parameters with the changes of the protein conformations, the protein CI2 (PDB code: 3CI2) was selected as a research object. 

**Figure 3:**
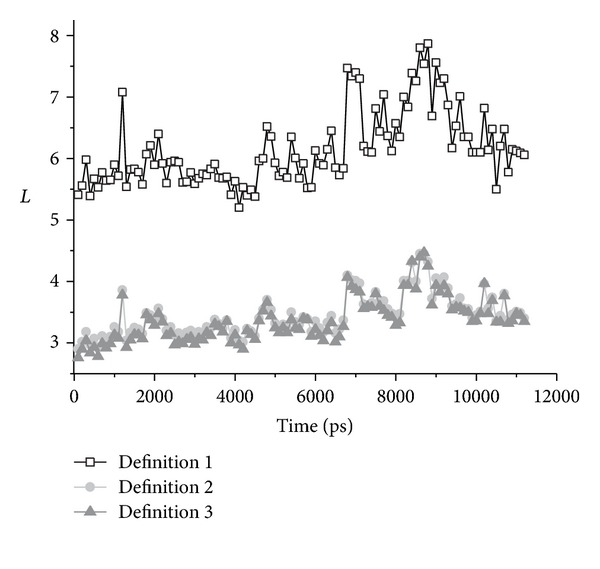
For the three definitions, the average shortest path length of the weighted amino acid networks of protein CI2 on the unfolding pathway.

With the MD program GROMACS 3.3 [20], the molecular dynamic (MD) simulation was performed at 498 K for 11.2 ns. The force field parameters used in this simulation were taken from GROMOS96 43a1 and the SPC/E water model was used. After the simulation, this protein will become unfolded, and most secondary structures will be depolymerized. However, the protein still keeps a random coil state. With this MD trajectory data, we extract the structures with an interval of 100 ps and then construct the weighted amino acid networks. On this unfolding pathway, along with the conformational changes, the change rule of the average shortest path length (short as: *L*) was analyzed. This change of *L* is used to represent the conformation change, and the results are shown in Figure 3. 

On the unfolding pathway, when the structure becomes looser, the average shortest path lengths of the weighted amino acid network become longer. Under a high temperature, with the unfolding of the protein, the hydrophobic core will be destroyed. In this process, the hydrophobic-hydrophobic link, which is important to the stability of the protein structures, will be broken. These hydrophobic-hydrophobic links all have a negative weight, and the distance of these links is less than 1. Therefore, while the hydrophobic core derogates, the shortest path length will rise more obviously. From Figure 3, we can see that the average shortest path length from definition 1 is more sensitive to the conformation change than that of the other two definitions.

### 3.4. The Application of the Amino Acid Network in Drug Design

In the process of drug action, many drugs take the related protein as their target. The structure and the dynamic of this target protein hold a very important role to the therapeutic effect of the drug. The residues located at the binding sites are crucial to the binding and the stability of the complex. These residues often are tightly packed and can provide a major part of the decrease of the binding free energy. They are often called as hot spots, and the central nodes of the amino acid network usually can be predicted as the hot spots [19, 29, 30]. With the support vector machine technology, a model is proposed for the prediction of the binding sites of heme protein [29]. This model contains three types of information: the first is the sequence information, the second is the geometry information of the structure, and the last one is based on some amino acid network parameter. Some scoring function based on the amino acid network also has been proposed for the protein docking [31–33]. Here, we take the immunosuppressant drug (FK506) binding protein—FKBP [34] as an example to show the application of amino acid network in the drug design. 

FKBP, or FK506 binding protein (PDB ID: 1FKF), is a immunophilins protein, which is involved in the immune response pathway and is used as a target for the immunosuppressant drug (FK506). Through the binding of FKBP with FK506, the signal transduction in T cells will be blocked, and then the normal immune system reaction will be interfered [35, 36]. 

**Figure 4:**
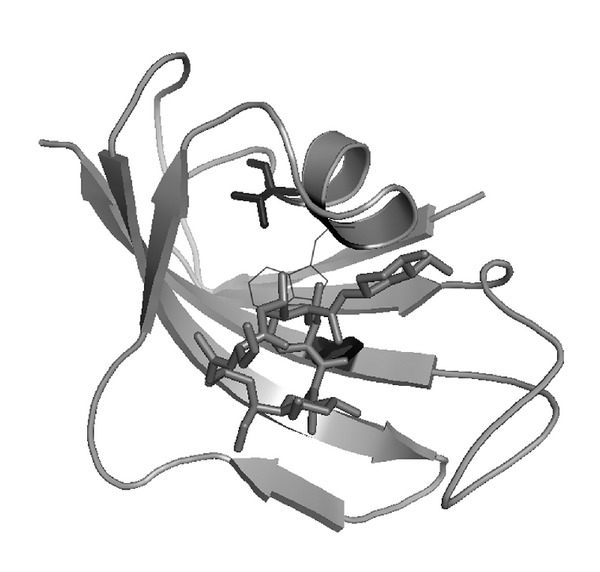
The structure of FKBP-FK506 complex (PDB ID: 1FKF). In this figure, the FK506 is shown with a stick model, and the FKBP is shown with a cartoon model. The Val^63^ and Phe^99^ are shown with stick model in black color. The Trp^59^ is shown with lines model.

Figure 4 shows the structure of the complex of FKBP with FK506.

With the structure, we can draw the detailed information about the complex that the binding sites contain which parts of the drug and which parts of the protein. We can find, as the structure showed above, that the *α* helix and the *β* sheet of the FKBP form a cavity, and the FK506 is binding with FKBP in this shallow cavity. For this structure, we construct its amino acid network and then calculate the related network parameter (betweenness) with the corresponding *Z*-score. In this work, only the *Z*-score value, which is greater than or equal to 3.0, is considered as a significant one, and the corresponding node will be discussed in the following parts [19]. For 1FKF, the calculating results show that there only two nodes get a higher *Z*-score value: Val^63^ and Phe^99^. At the same time, the contacts between the FKBP and FK506 are calculated. For FK506 holds a bigger volume than a residue, so, we use the atom contact between FK506 and FKBP. The Phe^99^ has ten atom contacts with the FK506, and these contacts are mainly due to the side chain of Phe^99^, which participates in assembling of the binding cavity with other residues. For Val^63^, although there is no direct interaction with FK506, it has nine atom contacts with Trp^59^, and Trp^59^ is interacted with FK506 through 20 atom contacts. The nodes with high *Z*-score value, for 1FKF, are either corresponding to the hot spot or to the residue which has a direct interaction with the ligand [19]. 

We also take the complex of fkbp12 with rapamycin (PDB ID: 1C9H [37] and 1FKB [38]) to calculate the *Z*-score value for every node of the amino acid network and to determine the contacts between the drugs with FKBP. The results also show that the node with high *Z*-score value either interacted directly with the drug or with nodes which is contacted directly with the drug. For these three proteins, the region from Phe^99^ to Val^101^ all contain a binding site with the drug. One is the Phe^99^ for 1FKF and 1FKB, and Val^101^ for 1C9H. On the other hand, when FK506 is binding to FKBP, we can find that the change of FKBP's structure is undersized, but the structural change of FK506 is large. So, we can deduce that the binding sites of FKBP with the related drug are spatial conserved. This useful information is helpful for the design of some new drugs, which has a better curative effect or less toxic than the FK506. 

## 4. Conclusion

A modified weighted amino acid network based on a self-consistent contact potential is proposed in this paper. This model contains two types of weight, one is the similar weight and the other is the dissimilar weight. By the analysis of the influence of different definitions of the distance based on the weights, it is revealed that the distance definition contains two types of weights is more reasonable. The average shortest path length has a significant linear correlation with the radius gyration of the molecule. For a set of 197 proteins, through the analysis of the network parameters of the weighted amino acid networks, it is found that the weighted amino acid network holds an obvious “small-world” property. Additionally, with the protein CI2 as an example, through the analysis of the changes of the weighted network parameters on the unfolding pathway, it is observed that the shortest path length of the weighted network will rise increasingly when the protein is unfolding. The highly central residues of the amino acid network play a key role in the binding of protein with drug. These central nodes either interacted directly with the drug or contacted with a residue which is interacted directly with the drug. In other words, for the interaction path between these central residues with the drug, at most, there is an interval between them.

This modified weighted network, which contains two types of weights, is more reasonable than the previous model. This work is helpful for the studies of the structure-function relationship and also is beneficial to the drug design.
